# Voluntary Movement Takes Shape: The Link Between Movement Focusing and Sensory Input Gating

**DOI:** 10.3389/fnhum.2018.00330

**Published:** 2018-08-17

**Authors:** Daniele Belvisi, Antonella Conte, Francesca Natalia Cortese, Matteo Tartaglia, Nicoletta Manzo, Pietro Li Voti, Antonio Suppa, Alfredo Berardelli

**Affiliations:** ^1^IRCCS Neuromed, Pozzilli, Italy; ^2^Department of Human Neurosciences, Sapienza University of Rome, Rome, Italy

**Keywords:** transcranial magnetic stimulation, motor surround inhibition, somatosensory temporal discrimination threshold, basal ganglia, voluntary movement

## Abstract

The aim of the study was to investigate the relationship between motor surround inhibition (mSI) and the modulation of somatosensory temporal discrimination threshold (STDT) induced by voluntary movement. Seventeen healthy volunteers participated in the study. To assess mSI, we delivered transcranial magnetic stimulation (TMS) single pulses to record motor evoked potentials (MEPs) from the right abductor digiti minimi (ADM; “surround muscle”) during brief right little finger flexion. mSI was expressed as the ratio of ADM MEP amplitude during movement to MEP amplitude at rest. We preliminarily measured STDT values by assessing the shortest interval at which subjects were able to recognize a pair of electric stimuli, delivered over the volar surface of the right little finger, as separate in time. We then evaluated the STDT by using the same motor task used for mSI. mSI and STDT modulation were evaluated at the same time points during movement. mSI and STDT modulation displayed similar time-dependent changes during index finger movement. In both cases, the modulation was maximally present at the onset of the movement and gradually vanished over about 200 ms. Our study provides the first neurophysiological evidence about the relationship between mSI and tactile-motor integration during movement execution.

## Introduction

Several mechanisms intervene in improving the accuracy of motor performance. In 2004, Sohn and Hallett found that voluntary activation of the first dorsal interosseous (FDI) muscle was associated with time-dependent inhibition of the nearby abductor digiti minimi (ADM), as tested by transcranial magnetic stimulation (TMS; Sohn and Hallett, 2004a). This phenomenon, called “motor surround inhibition” (mSI), resembles the surround inhibition previously described in the visual (Blakemore et al., 1970) and somatosensory (Tinazzi et al., 2000) systems. Previous neurophysiological studies investigating the possible mechanisms underlying mSI showed that primary motor cortex (M1) and cerebellum do not play a major role in mSI circuits (Beck and Hallett, 2011; Kassavetis et al., 2011; Sadnicka et al., 2013). Alternatively, Mink (1996) proposed that mSI might reflect the focusing activity performed by the basal ganglia. This hypothesis, however, has been never experimentally investigated.

The somatosensory temporal discrimination threshold (STDT) is a technique that measures the ability of a subject to recognize two stimuli as distinct in time (Conte et al., 2010, 2012, 2013, 2014). In a recent study, Conte et al. (2016) observed that STDT values significantly increased at the onset of a voluntary movement and the increase lasted about 200 ms. STDT modulation during movement execution reflects a basal ganglia-mediated sensory gating mechanism, needed to prioritize movement-related proprioceptive input during motor execution.

Both mSI and movement induced-STDT modulation intervene in shaping motor performance and are possibly related to each other. So far, however, no studies have investigated the possible relationship between the changes of mSI and STDT during motor execution. By comparing time dependent changes of the two mechanisms during the same time course we aimed to investigate the relationship between mSI and movement induced-STDT modulation.

To this purpose, we measured in healthy subjects mSI and movement induced-STDT modulation at the same time points during and up to 500 ms after the onset of the same motor task.

## Materials and Methods

### Participants

Seventeen healthy right-handed subjects (mean age 26 ± 8 years; nine men) participated in the study after giving their written informed consent. None of the participants had ever taken neuroleptic drugs or had a history of neuropsychiatric disorders, of neurosurgery or of metal or electronic implants. The local institutional review board approved all the experimental procedures that were conducted in accordance with the Declaration of Helsinki.

### Stimulation Techniques

For the assessment of mSI, single TMS pulses were delivered at rest and at the onset of the movement. We delivered TMS single pulses by using a monophasic Magstim 200 stimulator (Magstim Co, Whitland, Dyfed, UK). A figure-of-eight coil (external wing diameter: 9 cm) was placed tangentially over the left primary motor cortex (M1) in the optimal position to elicit motor evoked potentials (MEPs) in the right FDI and right ADM by delivering single TMS pulses. The intensity of the stimulation was adjusted to induce MEPs of approximately 1 mV in the resting ADM muscle. Single pulses were delivered at rest and 0 ms, 100 ms, 200 ms, 500 ms and 5 s after the onset of the movement.

To test STDT, we delivered couples of stimuli on the volar surface of little finger, starting with an interval between the two stimuli of 0 ms and progressively increasing the interval in 10-ms steps. We used a current stimulator (Digitimer DS7AH) to deliver square-wave electrical pulses through surface skin electrodes with the anode located 0.5 cm distally to the cathode. The stimulation intensity was defined by delivering a series of stimuli at an increasing intensity from 2 mA in 0.5 mA steps; the intensity used for STD testing was the lowest intensity the subject perceived in 10 out of 10 consecutive stimuli. The subjects verbally reported the number of stimuli perceived. The first of three consecutive intervals at which participants recognized the stimuli as temporally separate was considered the STDT. To check subjects’ attention level during the test and minimize possible perseverative responses, we included “catch” trials consisting of a single stimulus delivered randomly.

### Recordings

Electromyographic (EMG) activity was recorded from the right FDI and ADM muscles using a pair of Ag-AgCl surface electrodes in a belly-tendon montage. The ground electrode was placed above the styloid process of the right ulna. The EMG signal was amplified (1,000×) and band-pass filtered (bandwidth 20–2,000 Hz) with a Digitimer D360 amplifier (Digitimer Ltd, UK), digitized at a sampling rate of 5 kHz (CED 1401 laboratory interface; Cambridge Electronic Design, Cambridge, UK) and fed into a laboratory computer for storage and off-line analysis. Data were analyzed using SIGNAL software V5.08 (Cambridge Electronic Design, Cambridge, UK).

### Experimental Paradigm

#### Motor Surround Inhibition

**Figure 1:**
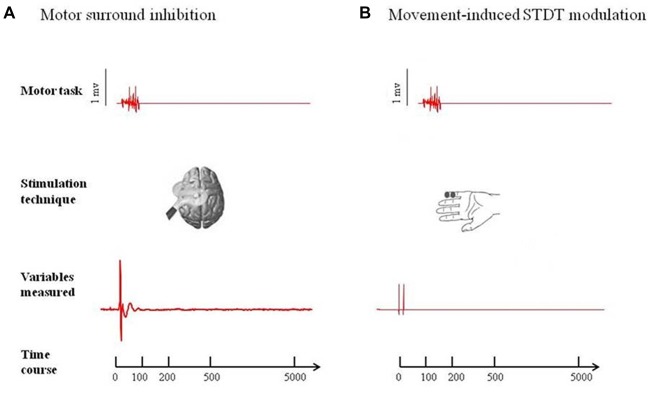
Experimental paradigms. **(A)** Motor surround inhibition (mSI): transcranial magnetic stimulation (TMS) single pulses were delivered during a brief flexion of the right index finger performed at 10% of the maximum electromyography (EMG) activity after a “go” signal. Motor evoked potentials (MEPs) were recorded from abductor digiti minimi (ADM; “surround muscle”) and first dorsal interosseous (FDI; “active muscle”) at 0 ms, 100 ms, 200 ms, 500 ms and 5 s after movement onset. **(B)** Movement induced-somatosensory temporal discrimination threshold (STDT) modulation: pairs of electrical stimuli were delivered on the volar surface of the right digiti minimi at 0 ms, 100 ms, 200 ms, 500 ms and 5 safter right index finger flexion performed at 10% of the maximum EMG activity after a “go” signal. Note that the magnetic stimuli for mSI and the electric stimuli for STDT were both triggered when EMG activity above 100 μV was detected in the right FDI (active muscle). mSI and movement induced-STDT modulation were evaluated in two distinct sessions performed on the same day.

At the beginning of the experiment, we measured the individual maximum EMG activity induced in the FDI (“active muscle”) by short-lasting index finger flexion. We then asked the subjects to perform brief movements at 10% of their maximum EMG activity while keeping their ADM (“surround muscle”) relaxed. Visual feedback of EMG activity from both muscles (FDI and ADM) was displayed on a screen in front of the subjects. Each subject attended a brief training session before the start of the experiment to ensure a consistent performance of the desired movement, with EMG activity in the ADM not exceeding 100 μV. To study mSI, TMS single pulses were delivered during a brief flexion of the right index finger after a “go” signal. The TMS single pulses for mSI were triggered (by using the SIGNAL “peritriggering function”) when EMG activity above 100 μV was detected in the right FDI and 20 MEPs were recorded at 0 ms, 100 ms, 200 ms, 500 ms and 5 s after movement onset. We considered the 5-s trial as the baseline (Figure 1).

#### Movement Induced-STDT Modulation

Subjects were asked to perform an index finger flexion at 10% of the maximum EMG activity (i.e., the same motor task used for testing mSI) and STDT was measured on the volar surface of the little finger. The STDT testing interval was progressively increased in 10-ms steps at each movement until when the subject recognized the two stimuli as sequential. The STDT was defined as the average of three STDT values, i.e., one for each block, and was entered in the data analysis. The electric stimuli for STDT were triggered (by using the SIGNAL “peritriggering function”) when EMG activity above 100 μV was detected in the right FDI (active muscle) and were delivered at 0 ms, 100 ms, 200 ms, 500 ms and 5 s after movement onset (Figure 1). mSI and movement induced-STDT were evaluated in two distinct sessions performed on the same day.

#### Movement Induced-STDT Modulation During Index Finger Abduction

To investigate whether the type of motor task might have influenced movement induced-STDT modulation, we measured the STDT on the volar surface of the little finger during an index finger abduction performed as widely and quickly as possible, i.e., with the same motor task used in the original protocol (Conte et al., 2016).

## Statistical Analysis

Peak-to-peak motor MEP amplitude for each trial was measured off-line and the average amplitude in 20 trials was calculated for each interval. mSI was expressed as the ratio of MEP amplitudes during peritriggered trials to MEP amplitudes in control trials [mSI (%) = (MEPcond/MEPtest) × 100]. To test the mSI at the baseline, we used a two-way repeated measures analysis of variance (ANOVA) with main factors MUSCLE (ADM vs. FDI) and interstimulus interval (ISI), i.e., the ISI between movement onset and electrical stimulation (ISI: six levels: baseline, 0 ms, 100 ms, 200 ms, 500 ms and 5 s after movement). We used a paired sample *t* test to compare MEPs amplitudes at the baseline with those at 0 ms, 100 ms, 200 ms and 500 ms after the onset of movement.

Shapiro-Wilks test to evaluate whether distribution was gaussian or not, and parametric or non-parametric tests were used accordingly. To analyze changes in STDT values during index finger flexion and during index finger abduction (control experiment) we used a repeated-measures ANOVA with factor ISI (ISI: five levels: baseline, 0 ms, 100 ms, 200 ms, 500 ms after movement). We used a one-way ANOVA to compare motor task-induced EMG activity (area under the curve [AUC]) of the FDI during movement induced-STDT with that present during mSI. The Greenhouse-Geisser correction was applied when needed. Tukey’s test was used for the *post hoc* analysis.

To measure intersubject variability of mSI and movement induced-STDT modulation, we used the coefficient of variation (COV), i.e., the ratio of the standard deviation to the mean. The COV was expressed as a percentage.

Pearson’s correlation coefficient was used to disclose any correlations between the STDT changes (expressed as STDT at “0 ms,” “100 ms,” “200 ms,” “500 ms”/baseline STDT ratio), ADM MEP changes (expressed as ADM MEPs at “0 ms,” “100 ms,” “200 ms,” “500 ms”/rest ADM MEP ratio) and FDI MEP changes (expressed as FDI MEPs at “0 ms,” “100 ms,” “200 ms,” “500 ms”/rest FDI MEP ratio).

All the results are reported at *p* < 0.05 after FDR correction for multiple comparisons. All values are expressed as mean ± standard deviations.

## Results

Shapiro-Wilks test showed that the data were normally distributed.

### Motor Surround Inhibition

**Figure 2:**
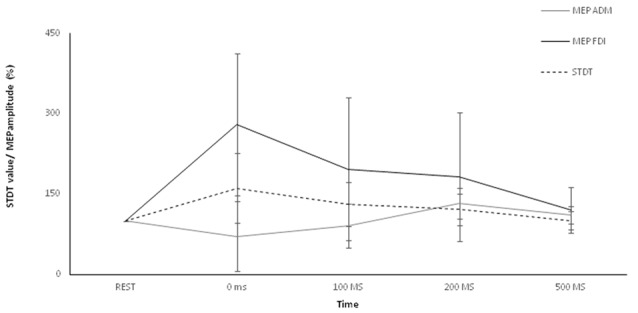
Main experiment. ADM and FDI MEP amplitude and STDT value changes index finger flexion. The extent of ADM/FDI MEP amplitude and STDT values change was maximal at the onset of the movement and gradually returned to the baseline values. X axis refers to the time course (baseline, 0 ms, 100 ms, 200 ms, 500 ms). Y axis refers to the percentage of ADM/FDI MEP amplitude and STDT values changes during movement. Vertical bars denote SD.

Two-way repeated measures ANOVA showed a significant factor MUSCLE (*F* = 59.7; *p* < 0.0001), factor ISI (*F* = 8.16; *p* < 0.0005) and a significant interaction MUSCLE × ISI (*F* = 8.71; *p* < 0.0001). The *post hoc* analysis showed that movement induced-changes in MEP amplitude in the ADM and FDI muscles were significantly different at 0 ms (*p* < 0.01), 100 ms (*p* < 0.01), 200 ms (*p* < 0.01), 500 ms (*p* < 0.01) and 5 s (*p* < 0.01) after the onset of movement. The paired sample *t* test showed that MEP amplitude of the ADM significantly decreased at 0 ms (*p* < 0.0001) and at 100 ms (*p* < 0.05), significantly increased at 200 ms (*p* = 0.01) and returned to baseline values 500 ms after movement onset (*p* > 0.05). The paired sample *t* test showed that MEP amplitudes of the FDI significantly increased at 0 ms (*p* < 0.0001), 100 ms (*p* = 0.006) and 200 ms (*p* = 0.011) though not at 500 ms after movement onset (*p* = 0.06; Figure 2).

### Movement Induced-STDT Modulation

Repeated-measures ANOVA showed a significant factor ISI (*p* < 0.0001). *Post hoc* tests showed that STDT values significantly increased when paired stimuli were delivered at movement onset (0 ms; *p* < 0.001), 100 ms (*p* = 0.008) and 200 ms (*p* < 0.03) after movement onset. The STDT values did not differ from baseline values at 500 ms (*p* > 0.05) and 5 s after the onset of movement (*p* > 0.05; Figure 2). No differences emerged in AUC when index finger flexion EMG activity was used for mSI and movement induced-STDT modulation (*p* > 0.05). The COV values of mSI and movement induced-STDT modulation are reported in Table 1.

**Table 1:** Coefficient of variation.

	ADM MEP	FDI MEP	STDT
Baseline	52%	47%	33%
0 ms	42%	59%	23%
100 ms	52%	57%	34%
200 ms	41%	51%	32%
500 ms	47%	43%	35%
5 s	49%	44%	30%

*Abductor digiti minimi (ADM), motor evoked potential (MEP), first dorsal interosseous (FDI), MEP and somatosensory temporal discrimination threshold (STDT) intersubject coefficient of variation (COV) at baseline and 0 ms, 100 ms, 200 ms, 500 ms and 5 s. The COV was calculated as the ratio of the standard deviation to the mean and was expressed as a percentage*.

### Movement Induced-STDT Modulation During Index Finger Abduction (Control Experiment)

**Figure 3:**
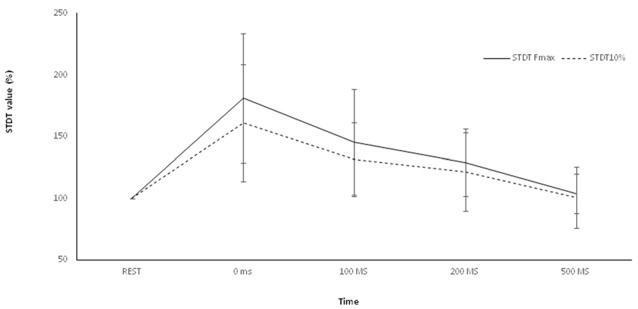
Control experiment. Movement-induced STDT value changes during index finger abduction perfomed at maximum force and index finger flexion performed at 10% of maximum force. STDT values changes showed a similar time course during the two motor tasks but the modulation was higher when the subjects perfomed an index finger abduxtion with the maximum force level. X axis refers to the time course (baseline, 0 ms, 100 ms, 200 ms, 500 ms). Y axis refers to the percentage of STDT values change during movement. Vertical bars denote SD.

Repeated-measures ANOVA showed a significant factor ISI (*p* < 0.00001). *Post hoc* tests showed that STDT values significantly increased at 0 ms (*p* < 0.0001), 100 ms (*p* = 0.006) and 200 ms (*p* < 0.02). No differences emerged in AUC when index finger flexion EMG activity was used for mSI and movement induced-STDT modulation (*p* > 0.05; Figure 3).

The COV values of mSI and movement induced-STDT modulation are reported in Table 1.

### Correlations

Pearson’s correlation coefficient revealed a significant correlation between movement induced-STDT changes and FDI MEP amplitude changes at 0 ms, 100 ms, 200 ms and 500 ms (*r* = 0.87; *p* = 0.01). By investigating the possible correlation between STDT changes during maximum force index finger abduction (control experiment) and FDI MEP amplitude changes, we also found a significant correlation (*r* = 0.98; *p* = 0.003). We found no significant correlation between STDT changes and ADM MEP amplitude changes.

## Discussion

In the present study, we observed that movement execution induces similar time-dependent changes in mSI and in movement induced-STDT modulation. The modulation in both cases was maximally present at the onset of movement and gradually vanished over a period of about 200 ms. In addition, movement-induced STDT changes correlated significantly with MEP amplitude facilitation of the FDI (i.e., the muscle involved in the motor task). By contrast, we did not detect any significant correlation between STDT changes and ADM MEP changes.

A strength of the present article is that we measured mSI and movement induced-STDT modulation by using the same motor task (index finger flexion performed with 10% of maximum force) and ensured that subjects exerted the same force level in the tasks by measuring FDI EMG activity. To avoid any methodological bias that might have led to our results being misinterpreted, we took several procedural precautions. During the baseline STDT testing, we delivered a “catch trial” to avoid attentional-related changes. To ensure that the baseline STDT value was reliable, we defined the STDT by repeating the measurement three times and calculating the mean of the three values. The different mSI and movement induced-STDT modulation sessions (0 ms, 100 ms, 200 ms, 500 ms and 5 s after the onset of movement) were administered in a randomized order. This allowed us to rule out the possibility that learning processes, fatigue mechanisms or changes in attention levels interfered with our findings.

In the present study, we observed that mSI and movement-induced STDT modulation have similar time-dependent changes. Our findings show that both mSI and movement induced-STDT modulation reach their maximum extent upon movement onset. Conversely, during the maintenance and tonic phases of the contraction, both mSI (Beck et al., 2008) and movement induced-STDT modulation (Beck et al., 2009) decay. However, while mSI is known to be stronger at low force levels (Beck et al., 2009), in the present study we observed that the extent of movement induced-STDT modulation is higher when force movement is maximum. Our interpretation is that at movement initiation, mSI is necessary for fine finger movements that require a low-force level (Beck et al., 2009). Conversely, movement induced-STDT modulation is greater in high-force level movements in which it is crucial to gate external inputs in favor of proprioceptive cues to optimize the scaling of movement parameters (Conte et al., 2016).

**Figure 4:**
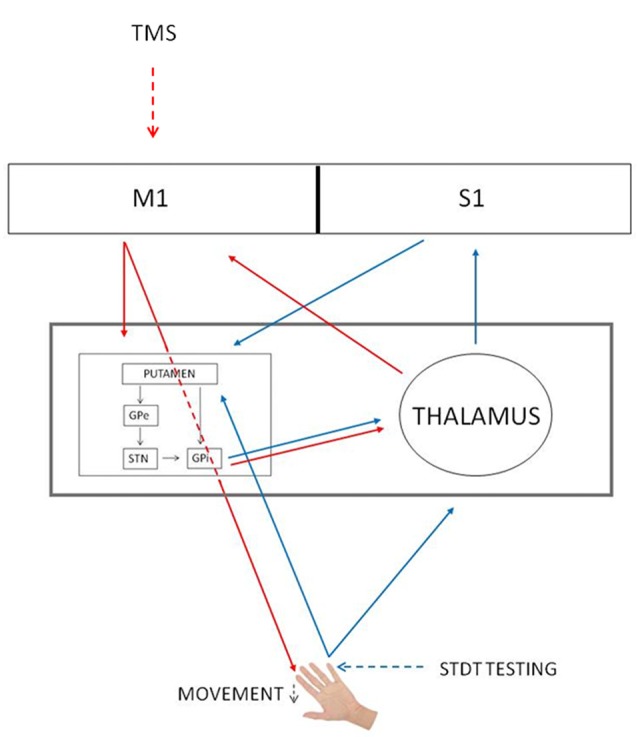
mSI and STDT gating circuits. Red arrows, mSI circuit. Blue arrows, STDT gating circuit. Black arrows, basal ganglia circuits. The gray rectangle shows that basal ganglia and thalamus underlie both mechanisms. GPe, globus pallidus externus; GPi, globus pallidus internus; M1, primary motor cortex; S1, primary somatosensory cortex; STN, subthalamic nucleus; TMS, transcranial magnetic stimulation.

Several studies have reported that the reduction of somatosensory evoked potentials at the onset of the movement is due to gating mechanisms occurring at cortical level (Starr and Cohen, 1985; Cohen and Starr, 1987; Jiang et al., 1991; Staines et al., 1997). Differently from these studies, in our previous article we have suggested that the modulation of STDT during movement depends on an interplay between the basal ganglia and the thalamus (Conte et al., 2016). We now speculate that subcortical structures (i.e., basal ganglia) contribute to mSI. In line with this hypothesis, Mink (1996) hypothesized that the inhibitory output of the basal ganglia selectively inhibits competing movements to prevent interference with the desired motor output (Beck and Hallett, 2011). Concurrently, basal ganglia gate non-movement-related external-generated signals while movement-related self-generated sensory information is being processed (Conte et al., 2016). In other words, the initiation of a voluntary movement induces a generalized motor excitation, which needs to be spatially and temporally shaped to produce an accurate movement. Therefore, mSI and movement induced-STDT modulation may reflect two different and complementary basal ganglia functions—respectively motor focusing and sensory gating—that are both designed to increase motor performance accuracy at the onset of movement (Figure 4).

This hypothesis fits well with previous evidence coming from previous studies on motor imagery. Aoyama et al. (2016) observed that there was no significant inhibitory effect on the MEP amplitude of the ADM during motor imagery of index finger flexion. Similarly, Conte et al. (2016) demonstrated that the STDT remained unchanged during a similar motor imagery task (Conte et al., 2016). Since motor imagery is believed to involve above all cortical pathways (Lacourse et al., 2005; Blefari et al., 2015; Pilgramm et al., 2016), the lack of mSI during motor imagery supports the hypothesis that mSI mainly relies on subcortical circuits.

Previous studies on patients with basal ganglia disorders provide further support for the involvement of basal ganglia circuits in mSI. mSI is abnormally reduced in patients with PD (Shin et al., 2007). Concordantly, recent evidence shows that movement induced-STDT modulation is altered in patients with PD and that dopaminergic treatment normalizes STDT gating in PD (Conte et al., 2017).

A support to the hypothesis that basal ganglia play a role in mSI comes from previous studies that have excluded an involvement of other central nervous system structures in mSI. Indeed, studies on mSI have ruled out any involvement of spinal mechanisms in mSI generation (Sohn and Hallett, 2004a; Belvisi et al., 2014) and previous studies have failed to demonstrate a relationship between mSI and other intracortical inhibition mechanisms, including long intracortical inhibition (Sohn and Hallett, 2004b), inter-hemispheric inhibition (Shin et al., 2009) and the cortical silent period (Poston et al., 2012). The data about the relationship between mSI and short intracortical inhibition (Stinear and Byblow, 2003; Sohn and Hallett, 2004a; Beck and Hallett, 2011) are contradictory and deserve further investigations. Previous neurophysiological studies demonstrated that cerebellar-brain inhibition is not functionally linked to mSI (Kassavetis et al., 2011) and that cerebellar modulation does not change mSI (Sadnicka et al., 2013) therefore suggesting that the cerebellum is unlikely to be involved in mSI.

A further new finding of our study is that movement induced-STDT modulation and FDI MEP facilitation during index finger flexion overlap in a time-dependent manner and positively correlate with each other. MEP facilitation during muscle contraction largely depends on the increasing excitability of spinal motoneurons and interneurons and partly on increasing descending activity (Di Lazzaro et al., 1998; Brum et al., 2016). Our findings show that subjects that display greater FDI MEP facilitation also display greater movement induced-STDT modulation, i.e., more efficient sensory gating. Bearing in mind that we have previously shown that movement induced-STDT modulation takes place at the supraspinal level (Conte et al., 2016), we suggest that the correlation between movement induced-STDT modulation and FDI MEP facilitation reflects descending volley activity. More efficient corticospinal tract activity may enhance sensory gating by boosting the motor flow directed from M1 to the striatum.

Although in the present study we observed that mSI and movement induced-STDT modulation show similar time dependent changes, we did not find a significant correlation between the extent of the modulation of two mechanisms during time. This is apparently contrasting to the hypothesis that mSI and movement induced-STDT modulation rely on the same neural structures. The lack of such a correlation is likely to depend on the extent of the two phenomena. mSI is a subtle mechanism required to shape fine finger movements that slightly reduces (by about 30%) surround muscle cortical excitability, with a high intersubject variability. By contrast, STDT modulation during voluntary movement reflects the function performed by the basal ganglia that filters irrelevant tactile stimuli for movement execution. STDT is largely modulated by movement execution (about 160%) and is a highly reproducible phenomenon. The different physiological features of mSI and movement induced-STDT may therefore explain the lack of correlation between the percentage changes in the two variables considered.

We acknowledge that our study has some limitations. The observation that mSI and movement induced- STDT modulation shows a similar time course only indirectly hints a relationship between the two physiological phenomena. In conclusion, the present study comparing two different physiological phenomena which are both time- and movement-related hints at a first neurophysiological evidence of a possible role played by the basal ganglia in mSI. We therefore propose that mSI and movement induced-STDT modulation may reflect two different and complementary basal ganglia functions that are both finalized to enhance the accuracy of voluntary movement.

## Author Contributions

DB and AC: conception and design of the study. DB, FNC, MT, NM, PLV and AS: acquisition of data. DB, AC, FNC, MT, NM, PLV and AS: analysis and interpretation of data. DB, AC and AB: drafting the article; critical revision for important intellectual content. AC and AB: final approval of the version to be submitted.

## Conflict of Interest Statement

The authors declare that the research was conducted in the absence of any commercial or financial relationships that could be construed as a potential conflict of interest.

## Abbreviations

ADM: abductor digiti minimi
AUC: area under the curve
COV: coefficient of variance
EMG: electromyography
FDI: first dorsal interosseous
ISI: interstimulus interval
MEPs: motor evoked potentials
mSI: motor surround inhibition
M1: primary motor cortex
STDT: somatosensory temporal discrimination threshold
TMS: transcranial magnetic stimulation.
